# Supplementary material for: Tetrazine-Induced Bioorthogonal Activation of Vitamin E-Modified siRNA for Gene Silencing
Source: Molecules. 2022 Jul 8;27(14):4377. doi: 10.3390/molecules27144377 (PMC9316517; doi:10.3390/molecules27144377)
Supplement: Supplementary file 1 [file molecules-27-04377-s001.zip › molecules-1804506-supplementary.pdf]

Supporting Information

Tetrazine-Induced Bioorthogonal Activation of Vitamin E-Modified siRNA for Gene Silencing

Xueli Zhang [1], Amu Gubu [1], Jianfei Xu [1], Ning Yan [1], Wenbo Su [1], Di Feng [1], Qian Wang [1] and Xinjing Tang [1,2]*

[1]State Key Laboratory of Natural and Biomimetic Drugs, School of Pharmaceutical Sciences and Chemical Biology Center, Peking University, No. 38 Xueyuan Rd., Beijing 100191, China;

[2]State Key Laboratory of Pharmaceutical Biotechnology, Nanjing University, Nanjing 210023, China

*Correspondence: xinjingt@bjmu.edu.cn

Synthesis of benzonorbonadiene linker phosphoramidite

Synthesis of compound 2

Pyrrole-2-carboxaldehyde (2.09 g, 22.0 mmol), N, N-diisopropylethylamine (2.84 g, 22.0 mmol) and 4-dimethyl-aminopyridine (0.27 g, 2.2 mmol) were dissolved in dichloromethane (100 mL). The solution was stirred for 5 minutes and acetyl chloride (1.88 g, 24.0 mmol) was then added. The reaction continued for 18 hours. The mixture solution was then diluted with dichloromethane (200 mL), washed with citric acid solution (50 mL, 10% w/v) once and water (100 mL) twice. The combined organic phase was dried over anhydrous Na_2SO_4. After concentration in vacuo, the residue was purified using silica gel column chromatography (petroleum ether: ethyl acetate =10:1) to obtain product as white solid (2.41 g, 17.6 mmol, yield 80%). ^{1}H NMR (400 MHz, Chloroform-d) δ 10.32 (s, 1H), 7.36 (dd, J=1.6, 2.8 Hz, 1H), 7.24 (dd, J=1.6, 4.0 Hz, 2H), 6.39 (t, J=3.2 Hz, 1H), 2.69 (s, 3H). ^{13}C NMR (101 MHz, Chloroform-d) δ 182.4, 169.1, 135.7, 126.6, 122.9, 112.9, 24.0.

Synthesis of compound 3

To a solution of compound **2** (0.96 g, 7 mmol) in tetrahydrofuran (50 mL), $Zn(BH_4)_2$ (0.76 g, 8 mmol), was added. The reaction was stirred for 30 minutes at 0℃ and was then quenched with acetic acid (10 mL) and water (10 mL). The resulting solution was extracted with dichloromethane and washed with water twice. The combined organic phase was dried over anhydrous Na_2SO_4. Upon concentration in vacuo, The residue was purified using silica gel column chromatography (petroleum ether: ethyl acetate =10:1) to give product as white solid (0.90 g, 6.4 mmol, yield 92%).^{1}H NMR (400 MHz, Chloroform-d) δ 7.10 (dd, J = 3.4, 1.6 Hz, 1H), 6.27 – 6.22 (m, 2H), 4.65 (s, 2H), 2.62 (s, 3H). ^{13}C NMR (101 MHz, Chloroform-d) δ 170.5, 135.6, 121.7, 114.7, 112.3, 57.9, 23.7.

Synthesis of compound 4

Compound **3** (0.70 g, 5.0 mmol) and imidazole (0.34 g, 5.0 mmol) were dissolved in N,N-dimethylformamide (5 mL). Tert-butyldimethylsilyl chloride (0.83 g, 5.0 mmol) was added to the solution. The reaction continued stirring for 3 hours at rt and was then quenched with saturated aqueous $NaHCO_3$. The resulting solution was extracted with ethyl ether. The combined organic phase was dried over anhydrous Na_2SO_4. After concentration in vacuo, the residue was purified using silica gel column

chromatography (petroleum ether: ethyl acetate =20:1) to obtain product as white solid (1.08 g, 4.3 mmol, 85%). ^{1}H NMR (400 MHz, Chloroform-d) δ 7.08 (ddt, J = 3.4, 1.6, 0.7 Hz, 1H), 6.34 (dq, J = 3.1, 1.5 Hz, 1H), 6.25 (t, J = 3.3 Hz, 1H), 4.96 (t, J = 1.0 Hz, 2H), 2.56 (s, 3H), 0.96 (s, 9H), 0.11 (s, 6H).^{13}C NMR (101 MHz, Chloroform-d) δ 169.1, 137.0, 120.3, 112.1, 111.6, 60.8, 25.9, 23.6, 18.4, -5.4.

Synthesis of compound 6

To a mixture of compound **5** (1.38 g, 10 mmol) and $NaHCO_3$ (1.09 g, 13 mmol) in a mixture of dichloromethane (10 mL) and methanol (5 mL), liquid bromine (1.60 g, 10 mmol) was slowly added under 4°C. After the addition, the reaction continued stirring for 30 minutes and was then quenched with water. The reaction was extracted with ethyl ether twice. The combined organic phase was dried over anhydrous Na_2SO_4. After concentration in vacuo, the residue was purified using silica gel column chromatography (petroleum ether: ethyl acetate=10:1) to obtain product as white solid (1.30 g, 6.0 mmol, yield 58%).^{1}H NMR (400 MHz, Methanol-d_4) δ 7.35 (s, 1H), 7.04 (s, 1H), 6.82 (d, J=2.0 Hz, 1H), 3.70 (t, J=5.2 Hz, 2H), 2.73(t, J=5.2 Hz, 2H). ^{13}C NMR (101 MHz, Chloroform-d) δ 152.2, 132.9, 129.5, 128.7, 115.7, 109.2, 62.8, 37.5. Mass (ESI, positive mode): calculated: 217.95, found: 218.83, 219.05.

Synthesis of compound 7

To a solution of compound **6** (1.30 g, 6 mmol) in anhydrous pyridine (10 mL), a solution of 4,4'-dimethoxytriphenylmethyl chloride (2.03 g, 6 mmol) in anhydrous pyridine (10 mL) was added and the reaction continued stirring overnight. Then, most of the pyridine was removed, and the residue was diluted with ethyl ether and washed with water for several times. The combined organic phase was dried over anhydrous Na_2SO_4. After concentration in vacuo, the residue was purified using silica gel column chromatography (petroleum ether: ethyl acetate: triethylamine =10:1:0.3) to obtain product as yellow oil (2.91 g, 5.6 mmol, yield 93%). ^{1}H NMR (400 MHz, Chloroform-d) δ 7.41 – 7.27 (m, 9H), 7.26 – 7.22 (m, 1H), 7.08 (dd, J = 8.2, 2.2 Hz, 1H), 6.96 (dd, J = 8.3, 2.1 Hz, 1H), 6.84 (dd, J = 9.2, 3.2 Hz, 4H), 3.82 (d, J = 1.9 Hz, 6H), 3.29 (q, J = 5.9 Hz, 2H), 2.82 (td, J = 6.7, 2.5 Hz, 2H). ^{13}C NMR (101 MHz, Chloroform-d) δ 158.4, 150.8, 145.2, 136.4, 133.1, 132.5, 130.0, 129.9, 128.2, 127.8, 126.7, 115.9, 113.1, 109.9, 86.1, 64.5, 55.2, 35.6. Mass (ESI, negative mode): calculated: 520.11, found: 519.14.

Synthesis of compound 8

Compound **7** (2.08 g, 4 mmol) and Hexamethyldisilane (5.86 g, 40 mmol) were dissolved in tetrahydrofuran (30 mL). The reaction was stirred for 12 hours at 65°C. Then, the reaction was concentrated and the residue was dissolved in tetrahydrofuran (20 mL) for next-step reaction without further purification. To the resulting solution, n-butyllithium in tetrahydrofuran (2.5 M, 3.12 mL) was added slowly at -84 °C. The reaction continued stirring for 30 minutes and was then quenched with saturated aqueous NH_4Cl. The solution was extracted with dichloromethane, and the combined organic phase was dried over anhydrous Na_2SO_4. After concentration in vacuo, the residue was purified by silica gel column chromatography (petroleum ether: ethyl acetate: triethylamine =10:1:0.3) to obtain the product as white solid (1.76 g, 3.4 mmol, yield 85%). ^{1}H NMR (400 MHz, Chloroform-d) δ 7.37 (d, J = 1.6 Hz, 1H), 7.35 (t, J = 1.4 Hz, 1H), 7.27 – 7.14 (m, 9H), 7.03 (dd, J = 8.1, 2.3 Hz, 1H), 6.81 – 6.74 (m, 4H), 6.58 (d, J = 8.1 Hz, 1H), 3.77 (s, 6H), 3.23 (t, J = 6.8 Hz, 2H), 2.78 (t, J = 6.8 Hz, 2H), 0.29 (s, 9H).^{13}C NMR (101 MHz, Chloroform-d) δ 158.9, 158.3, 145.3, 136.6, 136.1, 131.3, 131.1, 130.0, 128.2, 127.7, 126.6, 125.1, 114.3, 113.0, 86.0, 65.1, 55.2, 36.0, -0.9. Mass (ESI, negative mode): calculated: 512.24, found: 511.24.

Synthesis of compound 9

Compound **8** (1.76 g, 3.4 mmol) was dissolved in tetrahydrofuran (20 mL). 1,1'-sulfonyldiimidazole (1.26 g, 7.8 mmol) and cesium carbonate (0.64 g, 1.96 mmol) was added and the reaction was stirred for 12 hours.

After the removal of tetrahydrofuran, the residue was dissolved in ethyl ether and washed with saturated aqueous NH_4Cl. The combined organic phase was dried over anhydrous Na_2SO_4. After concentration in vacuo, the residue was purified using silica gel column chromatography (petroleum ether: ethyl acetate: triethylamine =10:1:0.5) to obtain product as white solid (1.28 g, 2.4 mmol, yield 72%). ^{1}H NMR (400 MHz, Chloroform-d) δ 7.94 (t, J = 1.1 Hz, 1H), 7.41 (d, J = 2.3Hz, 1H), 7.38 (t, J = 1.5 Hz, 1H), 7.35 – 7.30 (m, 2H), 7.27 – 7.18 (m, 8H), 7.15 (dd, J = 8.4, 2.3 Hz, 1H), 6.83 – 6.77 (m, 4H), 6.39 (d, J = 8.4 Hz, 1H), 3.80 (s, 6H), 3.28 (t, J = 6.3 Hz, 2H), 2.85 (t, J = 6.3 Hz, 2H), 0.37 (s, 9H). ^{13}C NMR (101 MHz, Chloroform-d) δ 158.4, 153.1, 145.1, 139.6, 137.5, 137.4, 136.2, 132.9, 131.8, 131.4, 129.9, 128.1, 127.7, 126.7, 118.4, 113.0, 86.0, 77.2, 64.2, 55.2, 36.1. Mass (ESI, negative mode): calculated: 642.22, found: 641.46, 677.45 (+Cl^-).

Synthesis of compound 10

Compound **4** (0.51 g, 2.0 mmol) and compound **9** (1.29 g, 2.0 mmol) were dissolved in anhydrous acetonitrile (20 mL), cesium fluoride (0.46 g, 3.0 mmol) was added and the reaction was stirred for 12 hours at 60°C. The reaction mixture was diluted with ethyl ether, washed with water twice and saturated aqueous NaCl once. The combined organic phase was dried over anhydrous Na_2SO_4. After concentration in vacuo, the residue was purified using silica gel column chromatography (petroleum ether: ethyl acetate: triethylamine =10:1:0.5) to obtain product as white solid (0.66 g, 1.0 mmol, yield 50%). ^{1}H NMR (400 MHz, chloroform-d) δ 7.77 – 7.52 (m, 1H), 7.39 – 7.33(m, 3H), 7.27 – 7.22 (m, 6H), 7.22 – 7.18 (m, 1H), 7.10 (s, 1H), 6.96 (s, 1H), 6.82 – 6.78 (m, 5H), 4.96 (d, J = 10.7 Hz, 1H), 4.69 (d, J = 10.4 Hz, 1H), 4.33 (t, J = 6.7 Hz, 1H), 3.80 (s, 6H), 3.24 (t, J = 6.9 Hz, 2H), 2.83 (t, J = 6.9 Hz, 2H), 1.89 (s, 3H), 0.97 (s, 9H), 0.21 (d, J =11.5 Hz, 6H). ^{13}C NMR (101 MHz, Chloroform-d) δ 167.7, 158.3, 145.2, 141.4, 136.5, 136.4, 136.3, 132.3, 130.9, 130.0, 129.0, 128.8, 128.1, 127.7, 126.6, 125.3, 113.0, 86.0, 65.6, 64.8, 60.9, 55.2, 36.8, 30.6, 25.8, 19.2, 18.2, -5.3, -5.5. Mass (ESI, positive mode): calculated: 675.34, found: 676.34, 698.27 (+Na^+).

Synthesis of compound 11

To a solution of compound **10** (0.66 g, 1 mmol) in tetrahydrofuran (10 mL), tetrabutylammonium fluoride (0.26 g, 1.0 mmol) was added. The reaction was stirred for 4 hours at rt. After concentration in vacuo, the residue was purified using silica gel column chromatography (petroleum ether: ethyl acetate: triethylamine =10:1:0.5) to obtain product as white solid (0.50 g, 0.9 mmol, yield 89%). ^{1}H NMR (400 MHz, chloroform-d) δ 7.35 (d, J = 1.2 Hz, 1H), 7.23 (m, 9H), 7.15 (d, J= 1.4 Hz, 1H), 7.06 (dd, J = 5.7, 2.6 Hz, 1H), 6.94 (d, J = 5.6 Hz, 1H), 6.87 (dd, J = 7.4, 1.5Hz, 1H), 6.82 – 6.78 (m, 4H), 5.47 (d, J = 2.6 Hz, 1H), 4.59 (dd, J = 7.2, 3.5 Hz, 2H), 3.80 (s, 6H), 3.25 (t, J = 6.8 Hz, 2H), 2.85 (t, J = 6.8 Hz, 2H), 2.02 (s, 3H). ^{13}C NMR (101 MHz, Chloroform-d) δ 164.8, 158.4, 148.5, 145.5, 145.2, 143.1, 136.9, 136.4, 136.3, 130.0, 128.1, 127.7, 126.6, 125.9, 121.7, 120.3, 113.0, 86.0, 66.9, 64.8, 58.1, 55.2, 53.4, 36.6, 22.3. Mass (ESI, positive mode): calculated: 561.25, found: 584.21 (+Na^+).

Synthesis of compound 12

Compound **11** (0.28 g, 0.5 mmol) and tetrazole (0.42 g, 0.6 mmol) were dissolved in anhydrous dichloromethane and was bubbled with nitrogen. 2-Cyanoethyl N, N-diisopropylchlorophosphoramidite (0.21 g, 0.9 mmol) was injected to the solution and the reaction continued stirring for 4 hours. The reaction mixture was concentrated in vacuo, and the residue was purified using silica gel column chromatography (petroleum ether: ethyl acetate: triethylamine =5:1:0.25) to obtain product as pale yellow oil (0.28 g, 0.4 mmol, yield 73%).^{1}H NMR (400 MHz, Chloroform-d) δ 7.37 (dd, *J* = 19.3, 7.0 Hz, 3H), 7.28 – 7.18 (m, 8H), 7.12 (d, *J* = 7.5 Hz, 2H), 7.01 (d, *J* = 10.9 Hz, 1H), 6.83 (d, *J* = 7.5 Hz, 1H), 6.79 (d, *J* = 8.8 Hz, 4H),

5.39 (s, 1H), 4.86 (t, J = 49.8 Hz, 2H), 3.92 (dd, J = 12.6, 6.3 Hz, 2H), 3.80 (s, 6H), 3.71 (dd, J = 16.6, 6.7 Hz, 2H), 3.24 (t, J = 6.8 Hz, 2H), 2.83 (t, J = 6.7 Hz, 2H), 2.64 (d, J = 6.2 Hz, 2H), 1.90 (s, 3H), 1.28 – 1.22 (m, 12H). ^{31}P NMR (162 MHz, Chloroform-d) δ 148.77.

General method for oligonucleotide synthesis and purification

Oligonucleotides in this study were synthesized on ABI394 DNA/RNA synthesizer using standard RNA synthesis procedure. Benzonorbonadiene linker phosphoramidite and vitamin E phosphoramidite were sequentially modified to the 5′ terminal of oligonucleotides. All the oligonucleotides were cleaved and deprotected from solid CPG with the mixture solution of aqueous ammonium hydroxide (28 %, 300 μL) and aqueous methylamine (40%, 300 μL) overnight at room temperature. The solution was concentrated in vacuo and the residue was redissolved in DMSO (150 μL). Triethylamine trihydrofluoride was added and the reaction was allowed to votex for 2 hours at 65°C to remove the TBDMS protecting group of 2′ hydroxyl group. Sodium acetate (3M, 30 μL) and anhydrous ethanol (900 μL) were added to the solution. The resulting solution was stored in −30 °C freezer for 3 hours to precipitate the oligonucleotides. Then, the mixture was centrifugated (4 °C, 14000×g, 15 min) and the supernatant was discarded. The precipitates were washed with cold ethanol solution (75%, 500 μL) and collected for further HPLC purification. The modified oligonucleotides were purified using HPLC (Waters 2695 system) with reverse-phase HPLC column (XBridge®Oligonucleotide BEH C18, 2.5 μm,10 mm × 50 mm) according to the following procedure (A: 0.05 M triethylammonium bicarbonate buffer, TEAB, pH 8.5; B: acetonitrile).

Table S1. Condition of HPLC purification

**Time(minutes)**	**Rate of flow(mL/min)**	**A%**	**B%**
0	1.00	100.0	0.0
32.00	1.00	36.0	64.0
33.00	1.00	0.0	100.0
35.00	1.00	0.0	100.0
36.00	1.00	100.0	0.0
40.00	1.00	100.0	0.0

Cell toxicity study of tetrazine by SRB assay

HepG 2 cells (293A cells) were cultured in DMEM supplemented with 10% FBS, 100 μg/mL penicillin and 100 μg/mL streptomycin (37 °C, 5% CO_2). Cells were seeded in 96-well plates with a density of 8000 cells/well and were further cultured for 24 hours. Various concentrations of tetrazine in DMSO were added to cells for further 24 h incubation. The DMEM medium was removed and 100 μL 10% trichloroacetic acid was added to each well. After another 1 h incubation at 4 °C, the trichloroacetic acid solution was removed, and the wells were washed with deionized water 4 times and then dried. A total of 100 μL Sulforhodamine B (SRB) solution (4 mg/mLl) was added for 30 min incubation and the SRB solution was then removed. The wells were washed with 1% acetic acid 4 times and dried. A total of 100 μL Tris solution (10 mM, pH 10.5) was added to the wells. The 96-well plates were read for optical density at 540 nm by FlexStation 3 Benchtop Multi-Mode Microplate Reader (Molecular Devices).

Table S2. The sequences and molecule weights of oligonucleotides in this study

Target	Name	Sequence	Calcd.	Found
GFP	VBASG	VB-GUUCACCUUGAUGCCGUUCTT	7375.1	7376.6
	SG	GAACGGCAUCAAGGUGAACTT	--	--
Eg5	VBASE	VB-AUAGACUUCAUCCUUGUUGTT	7381.3	7384.8
	VASE	V- AUAGACUUCAUCCUUGUUGTT	7060.2	7063.5
	SE	CAACAAGGAUGAAGUCUAUTT	--	--

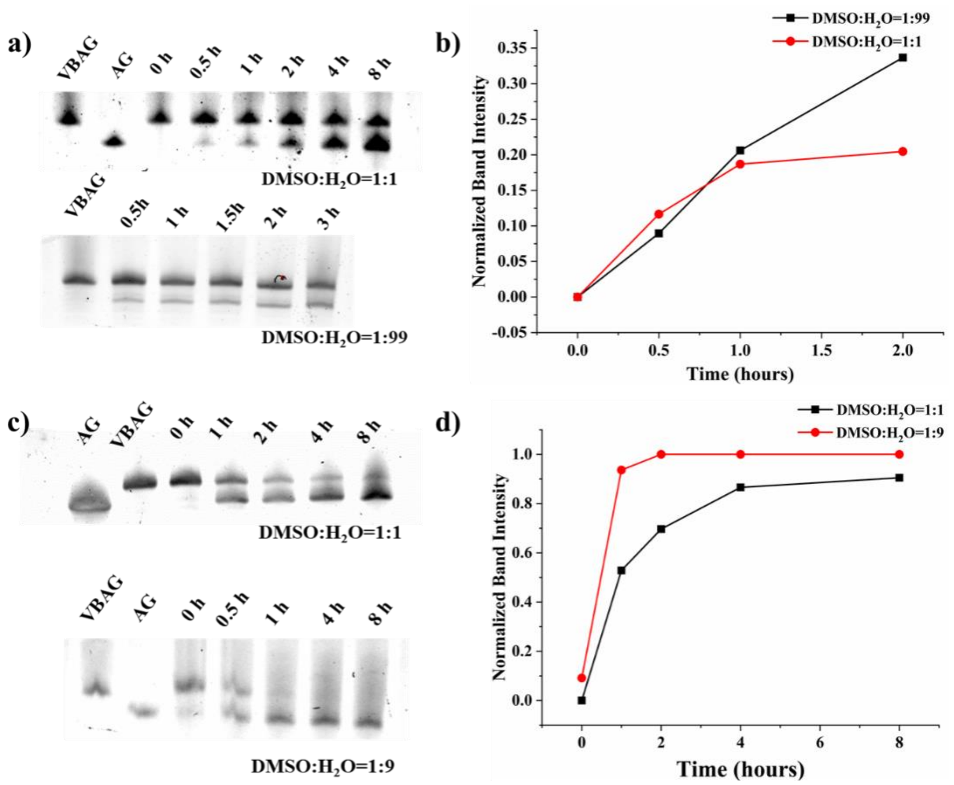

Figure S1. Bioorthogonal reaction rate of vitamin E-benzonorbonadiene caged RNA (VBAG) with tetrazine. a, b) The PAGE gel and quantitative analysis of gel band intensity analysis of caged oligonucleotides with the addition of 1 mM tetrazine in different ratios of DMSO and H_2O. c, d) The PAGE gel and quantitative analysis of gel band intensity analysis of caged oligonucleotides with the addition of 10 mM tetrazine The in different ratios of DMSO and H_2O. Image J software, the uncaged RNA band intensity was normalized to the sum of band intensities of caged and uncaged RNAs after quantification of Image J software.

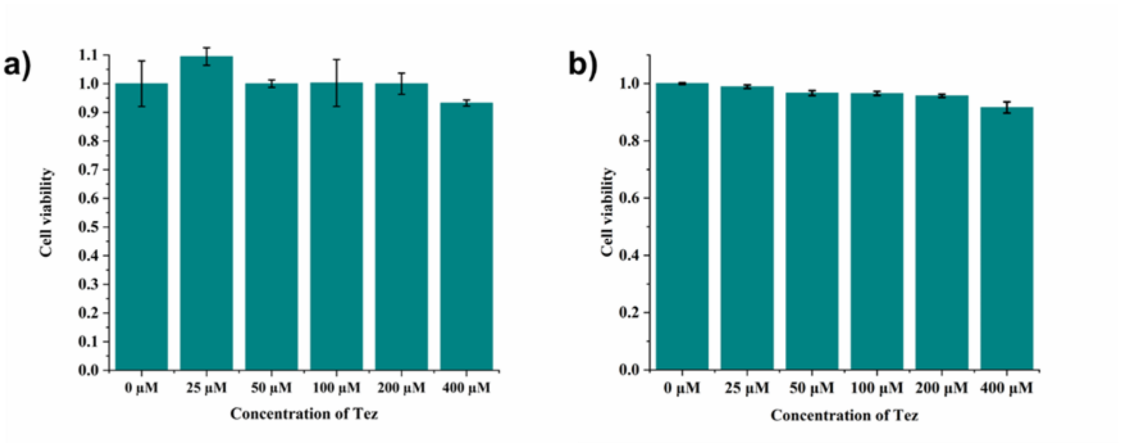

Figure S2. Cell toxicity of tetrazine to a) HepG 2 cells and b) 293A cells. At least three independent experiments were performed. Error bars represent standard deviations.

NMR of compound **2** to compound **11**

^{1}H NMR of **2**

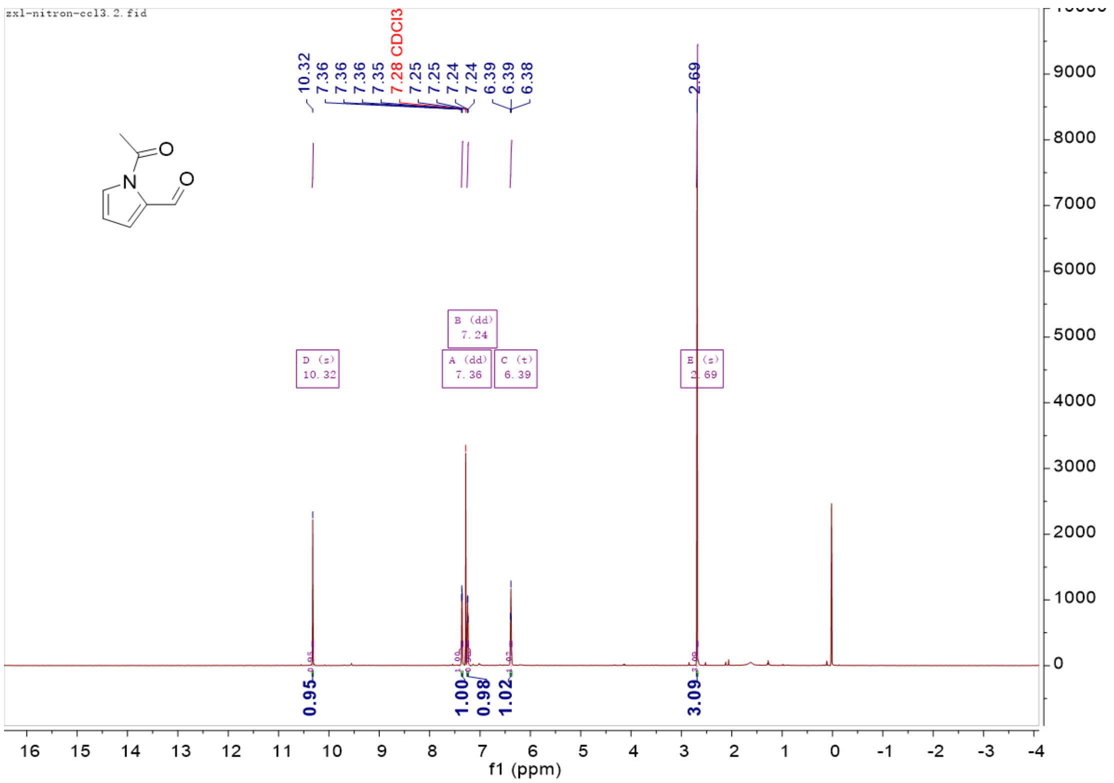

^{13}C NMR of **2**

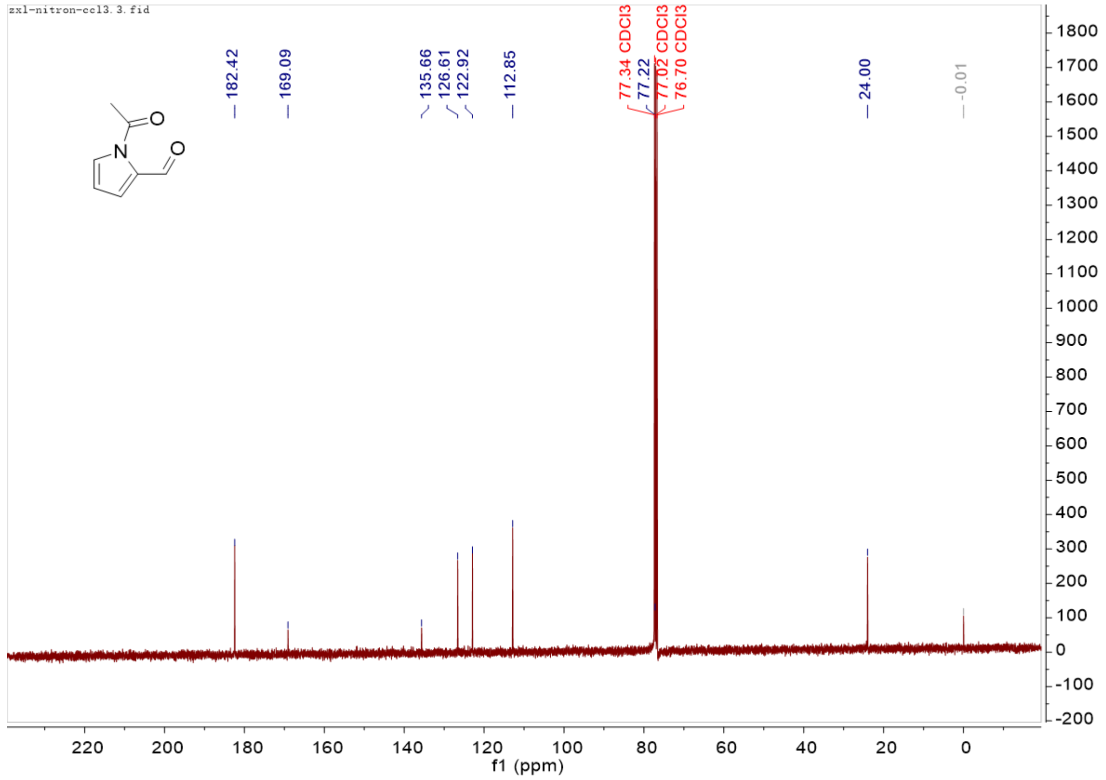

^{1}H NMR of **3**

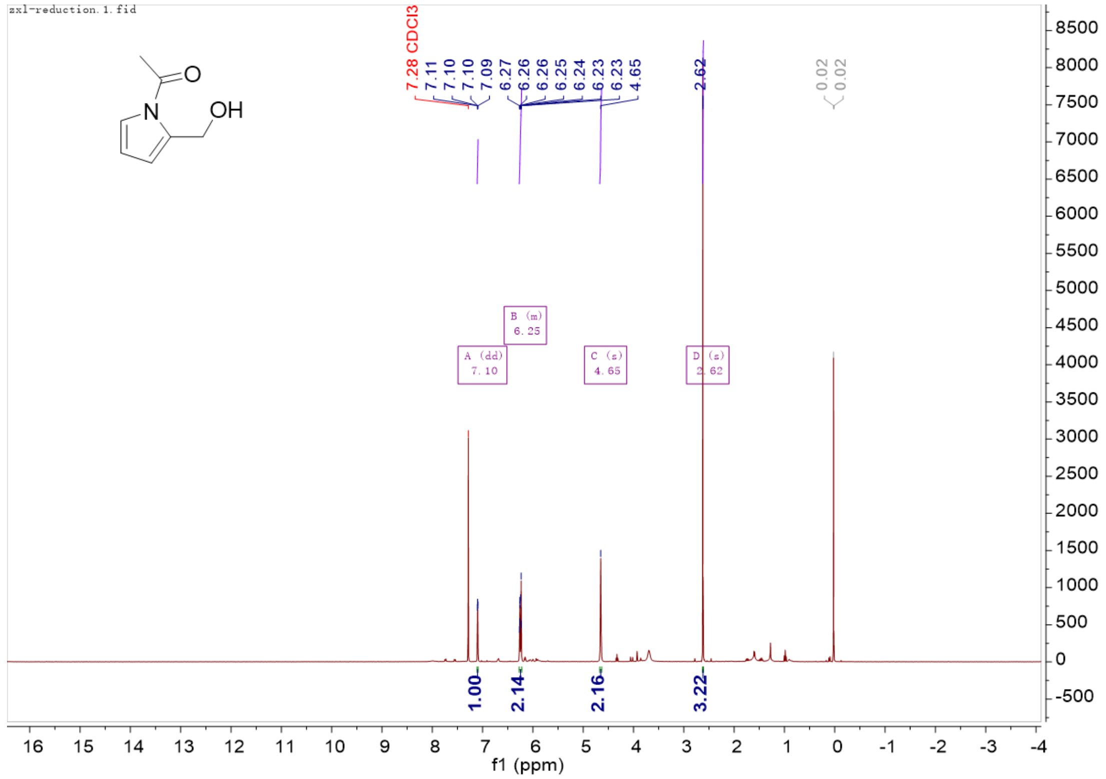

^{13}C NMR of **3**

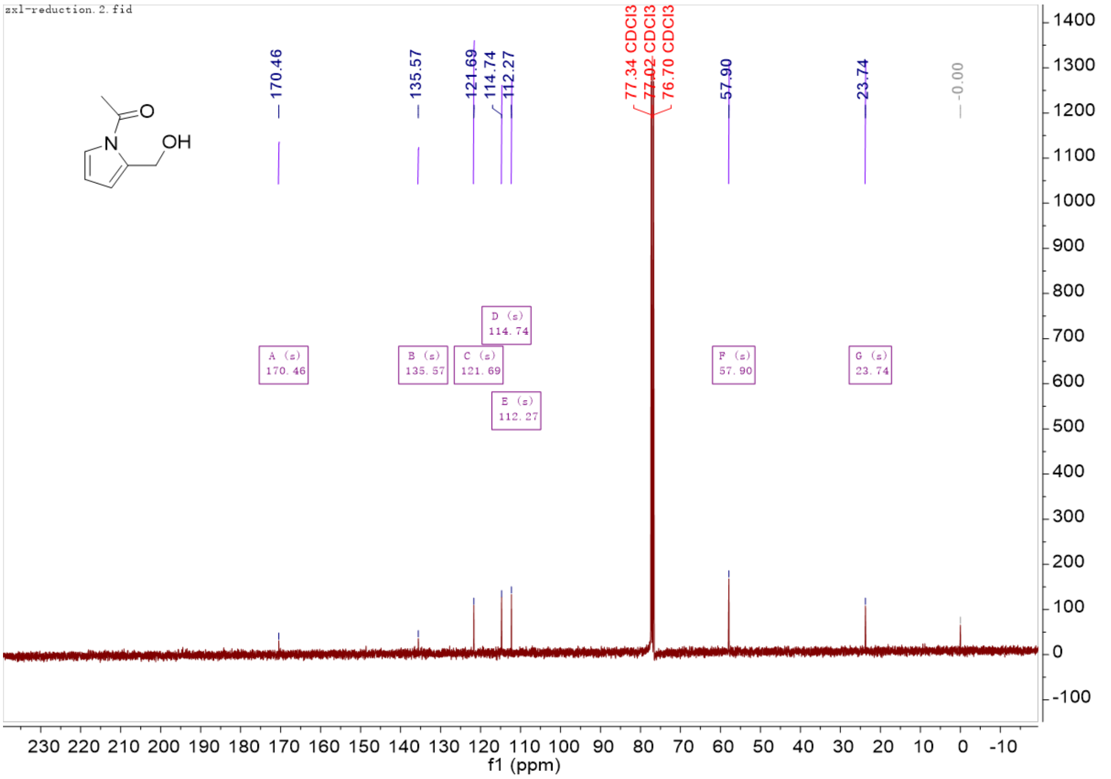

^{1}H NMR of **4**

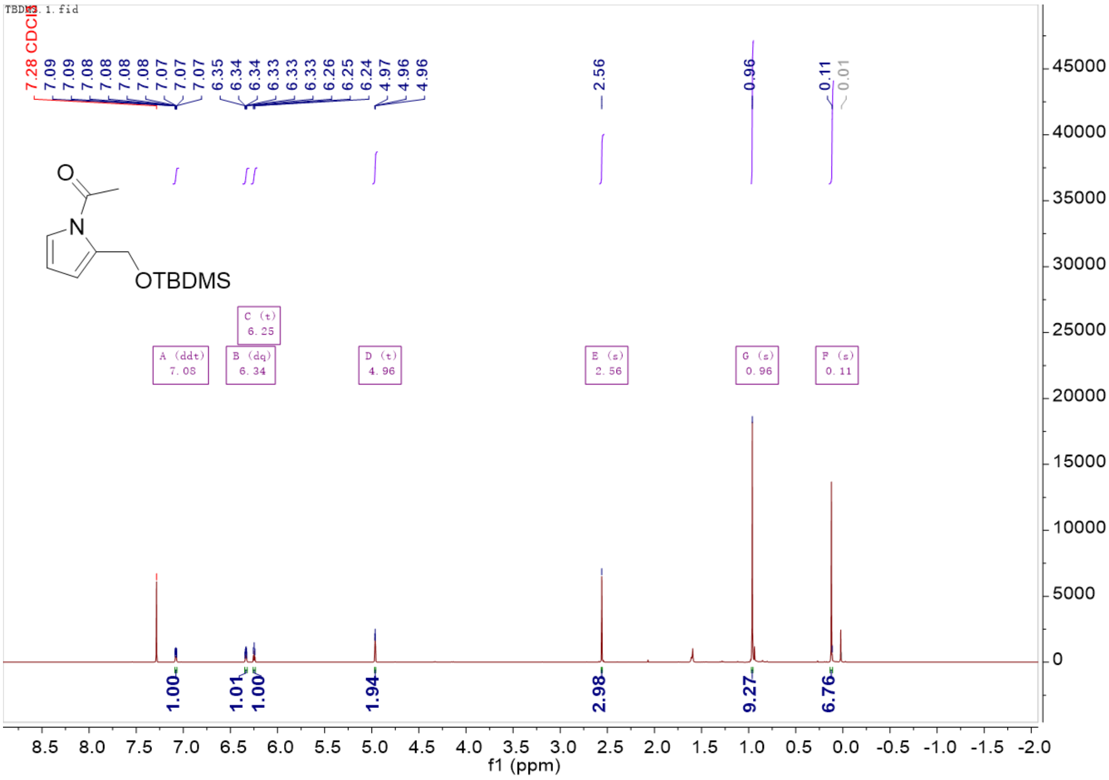

^{13}C NMR of **4**

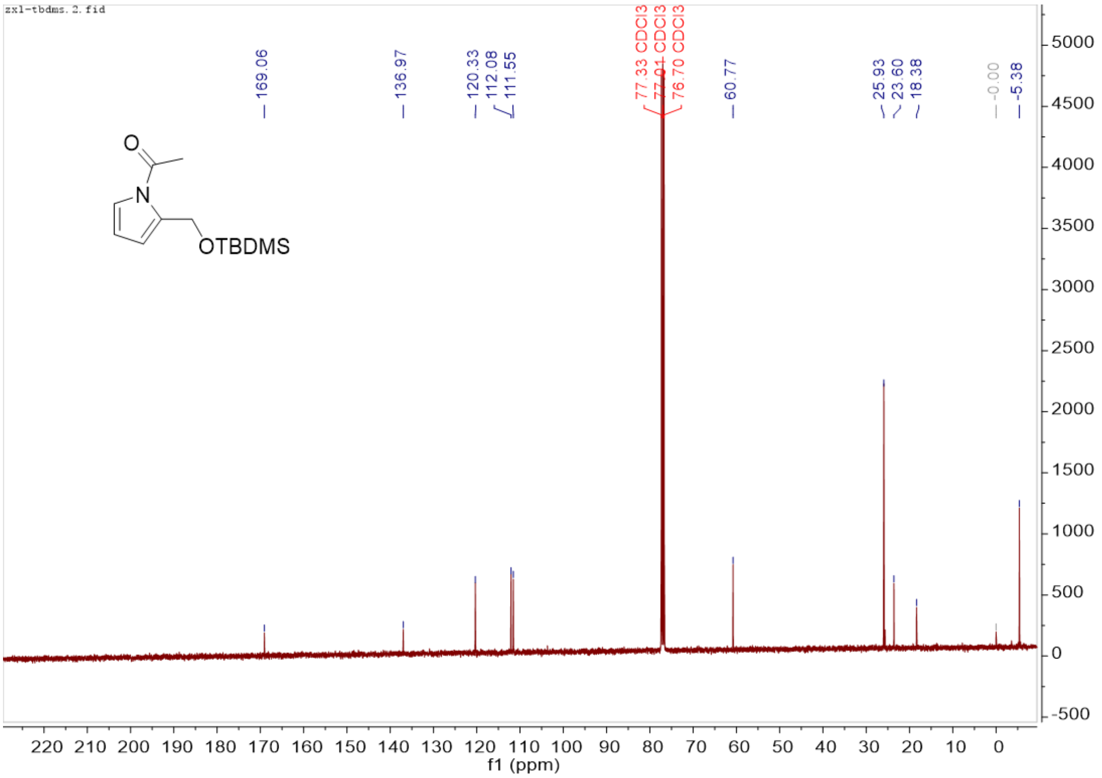

^{1}H NMR of **6**

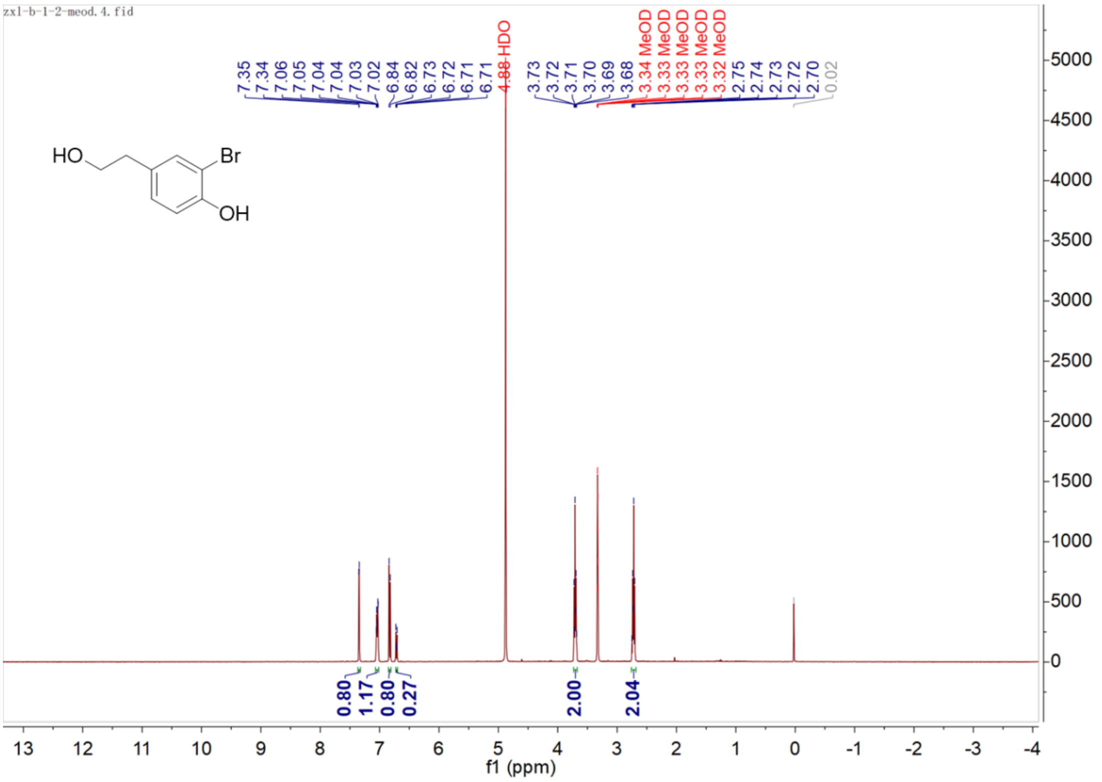

^{13}C NMR of **6**

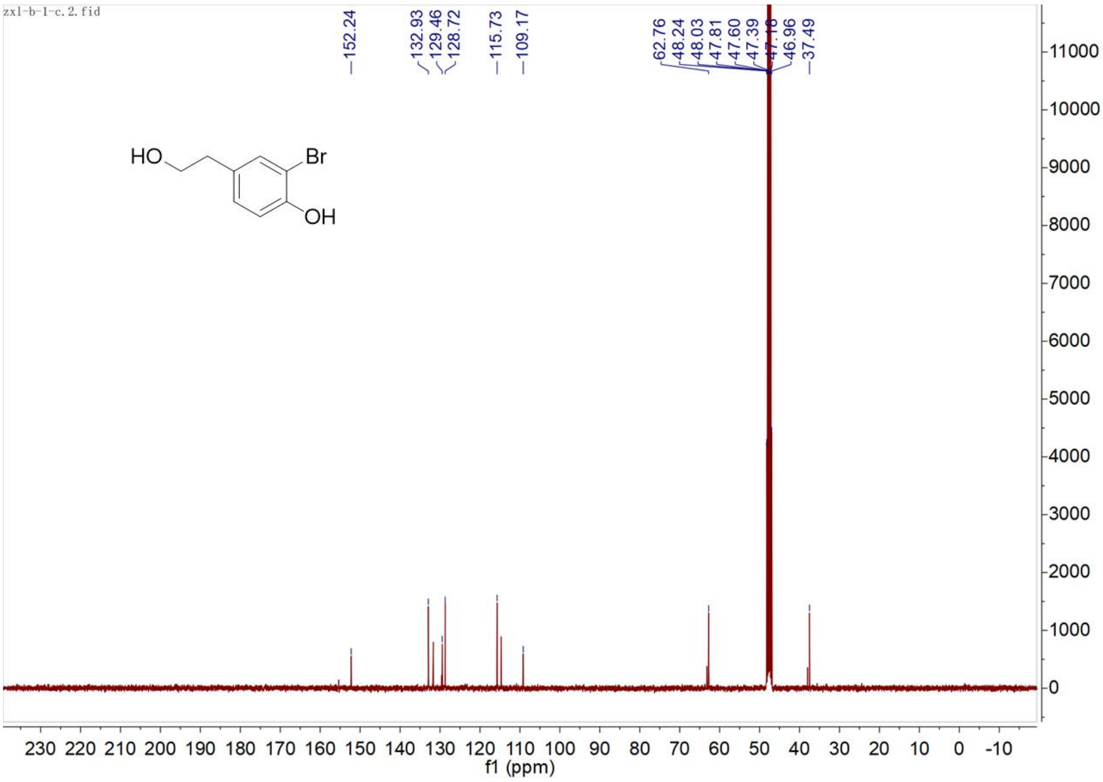

^{1}H NMR of **7**

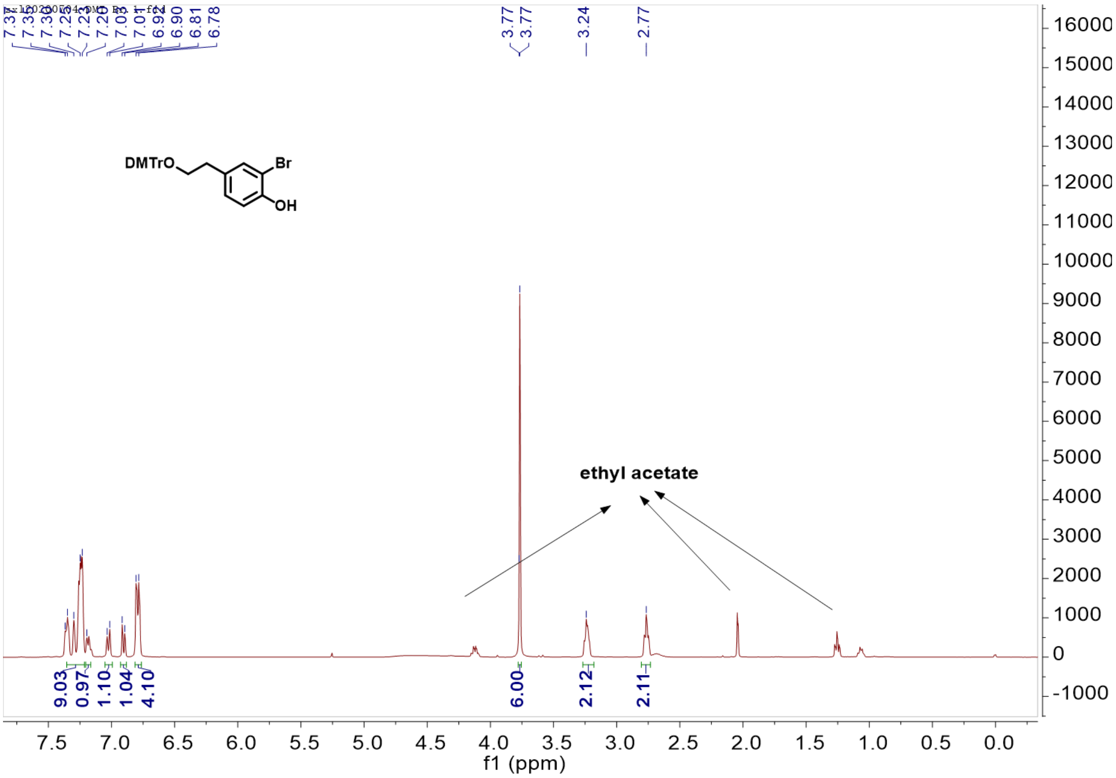

^{13}C NMR of **7**

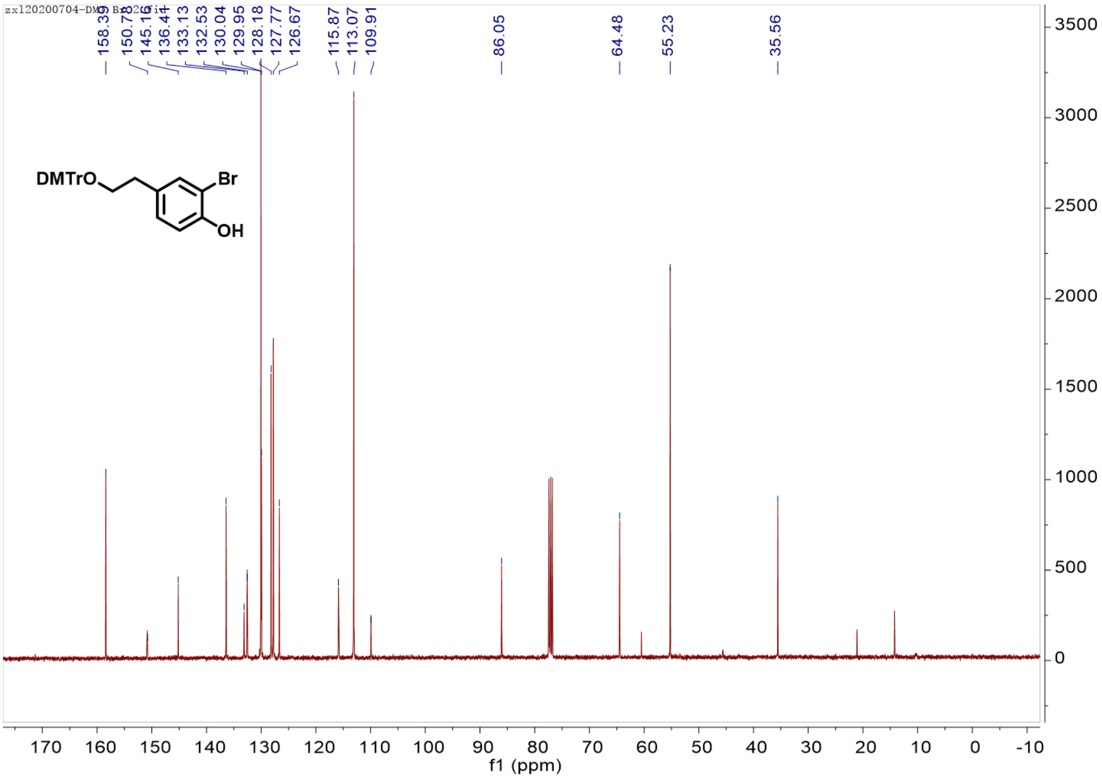

^{1}H NMR of **8**

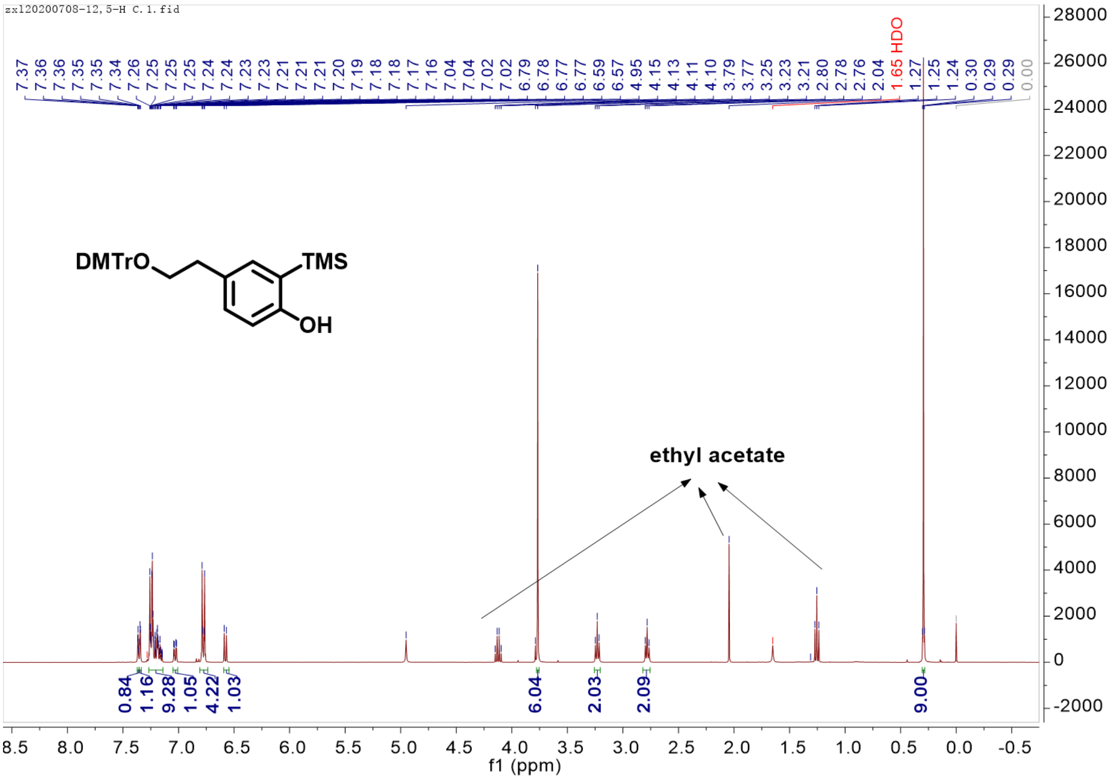

^{13}C NMR of **8**

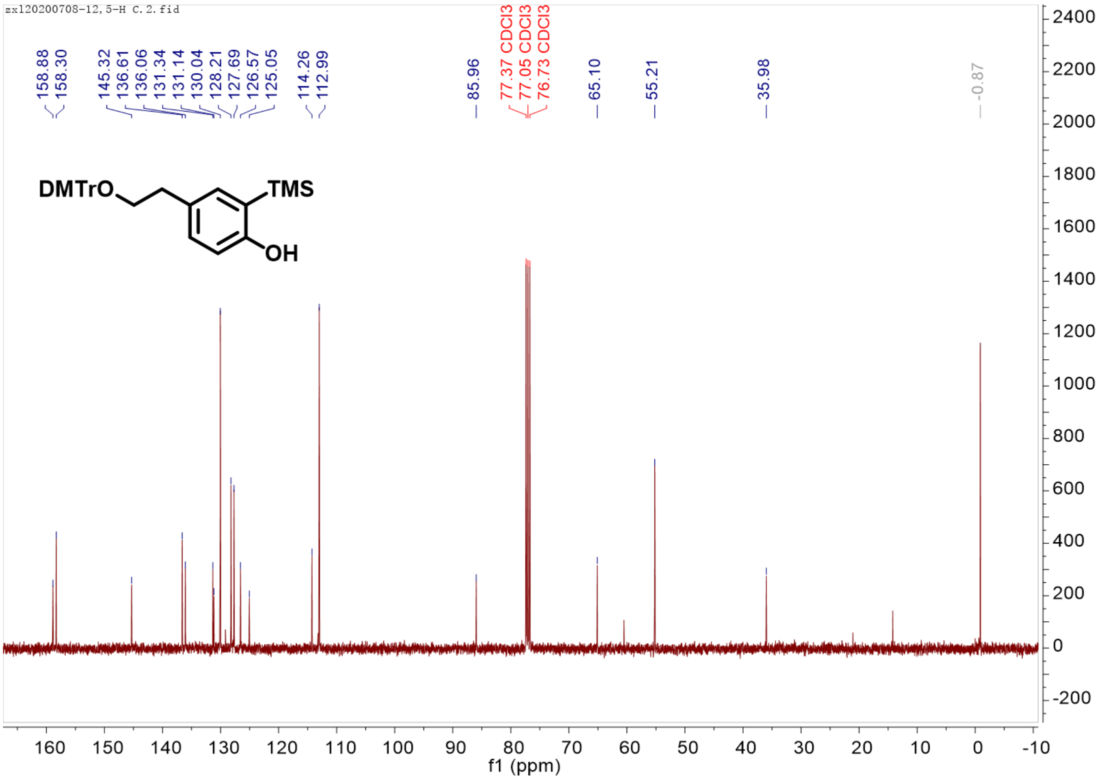

^{1}H NMR of **9**

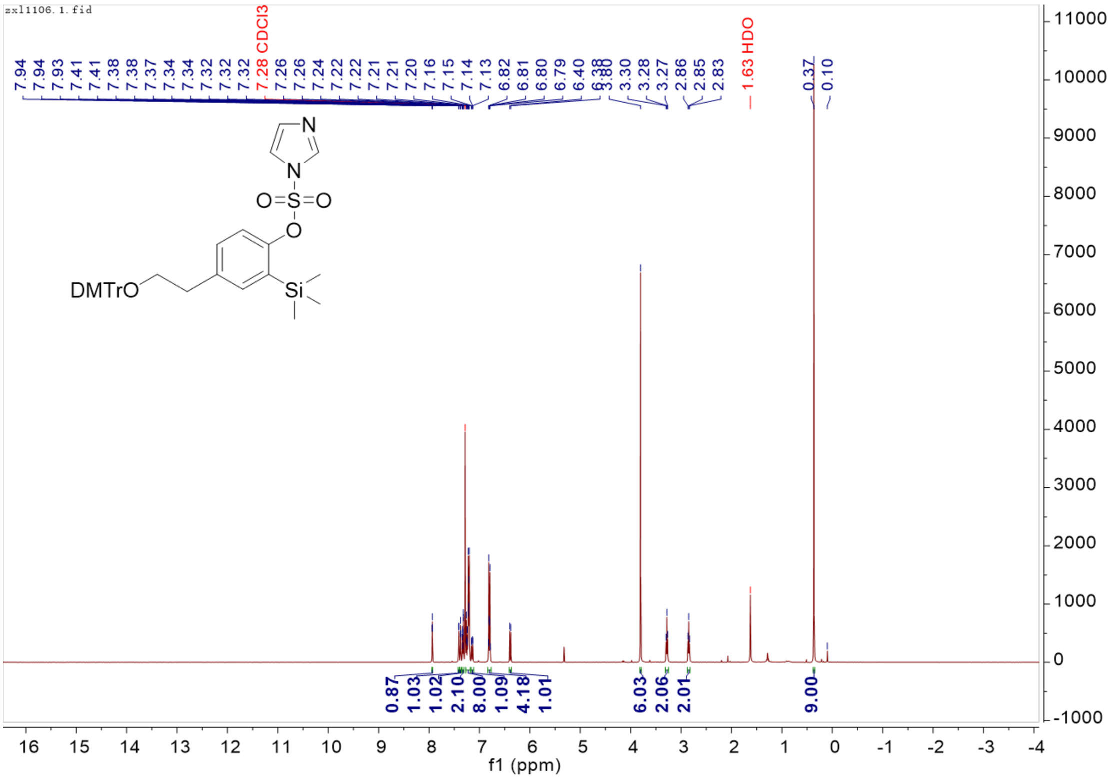

^{13}C NMR of **9**

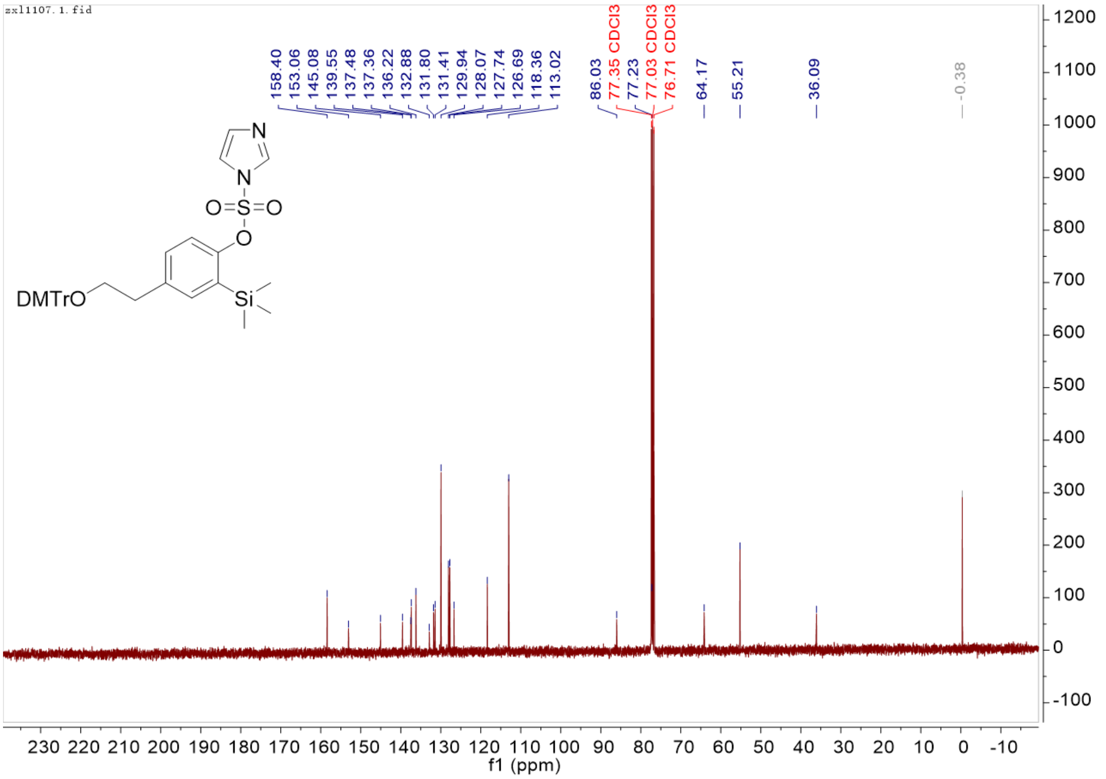

^{1}H NMR of **10**

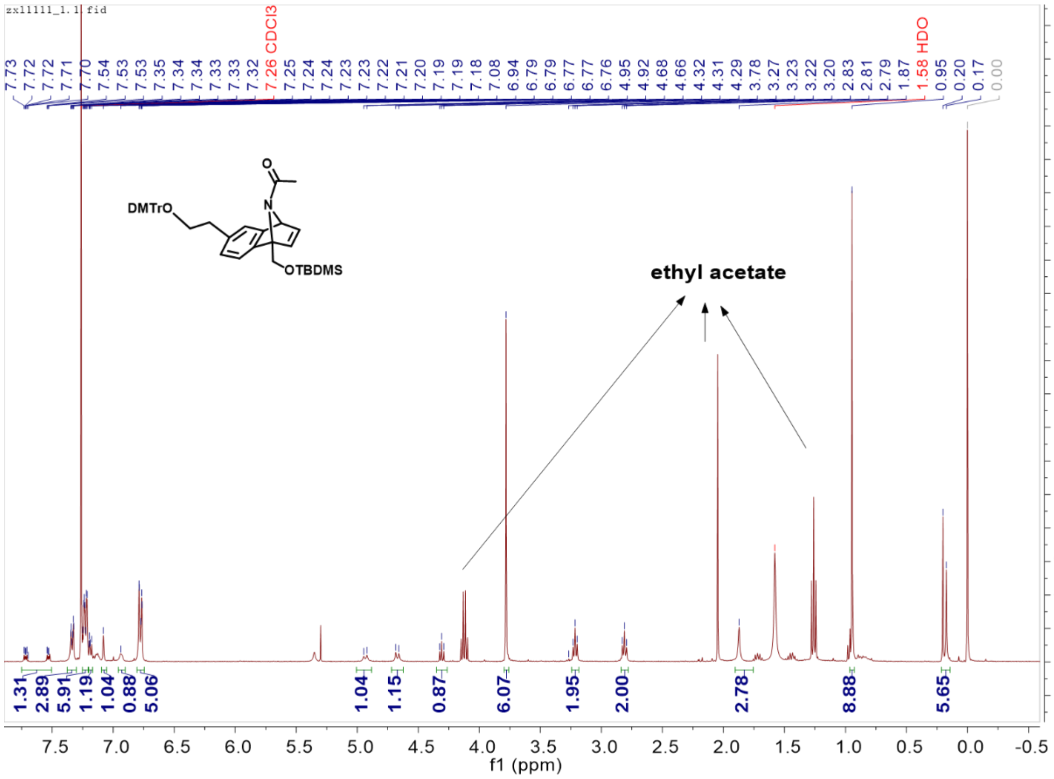

^{13}C NMR of **10**

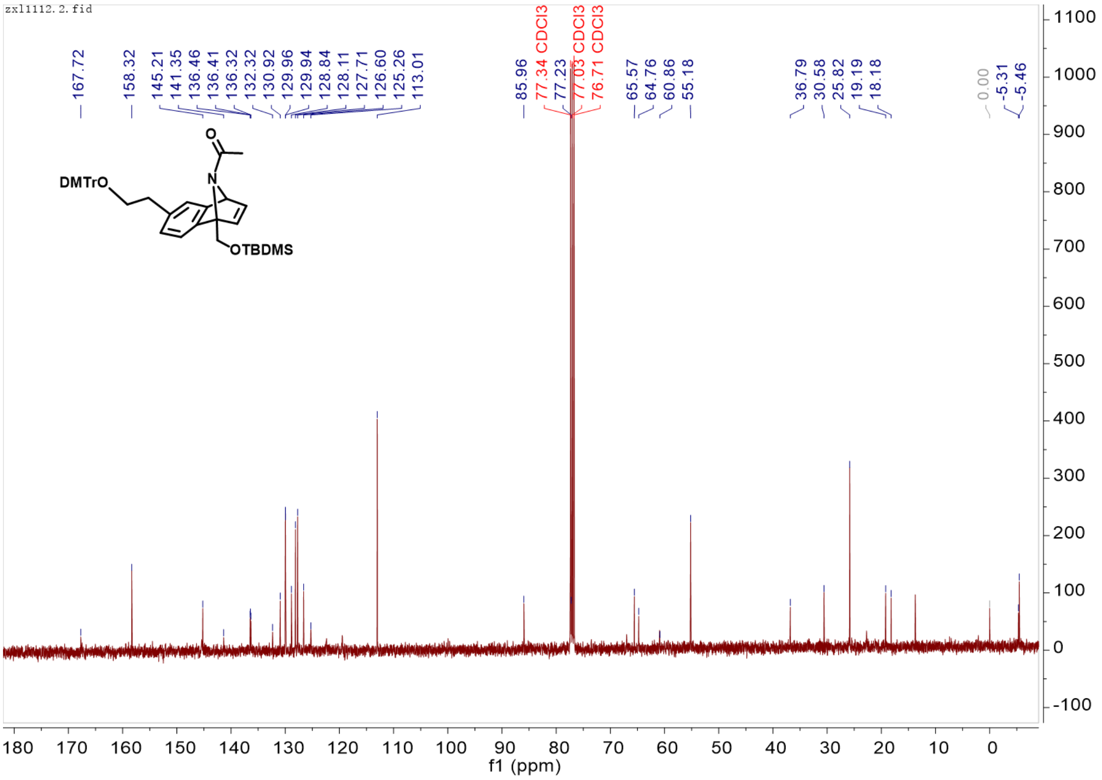

^{1}H NMR of **11**

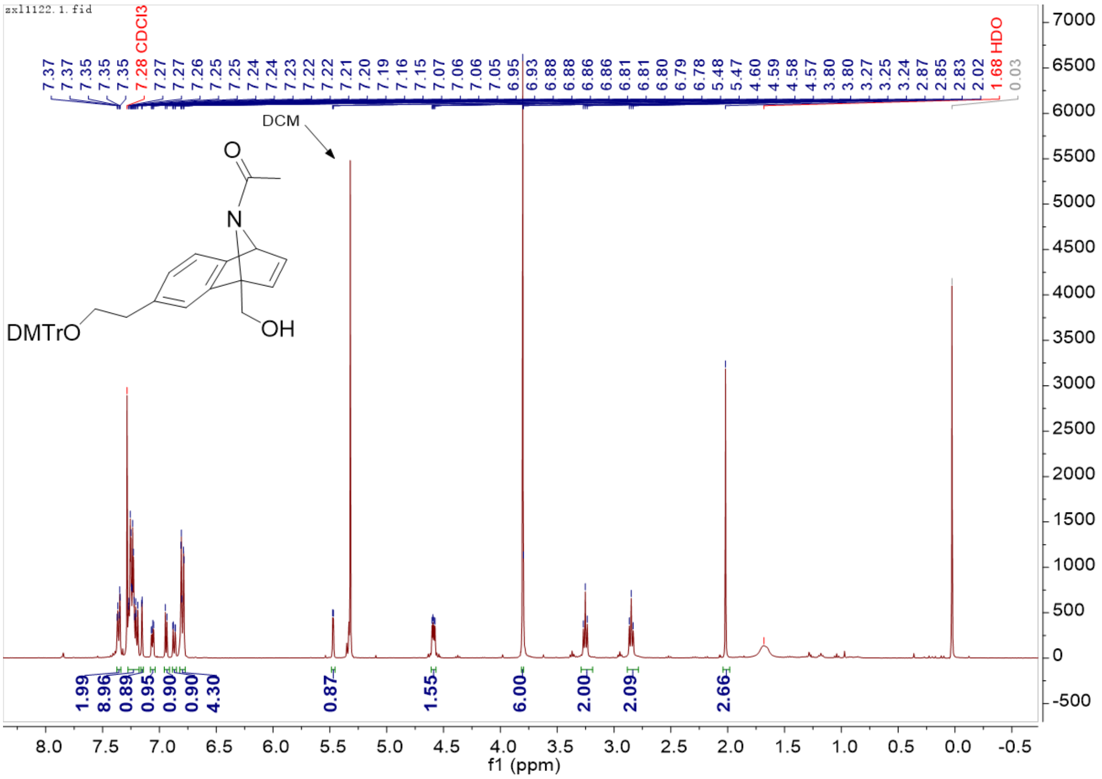

^{13}C NMR of **11**

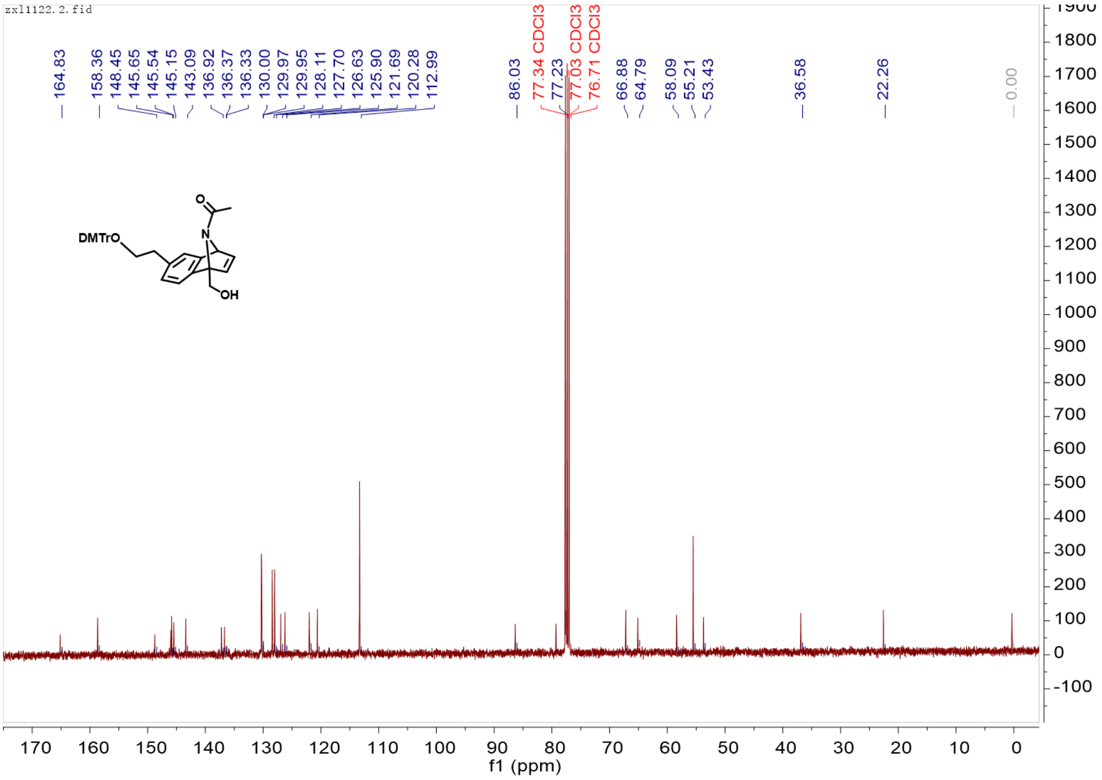

^{1}H NMR of 12

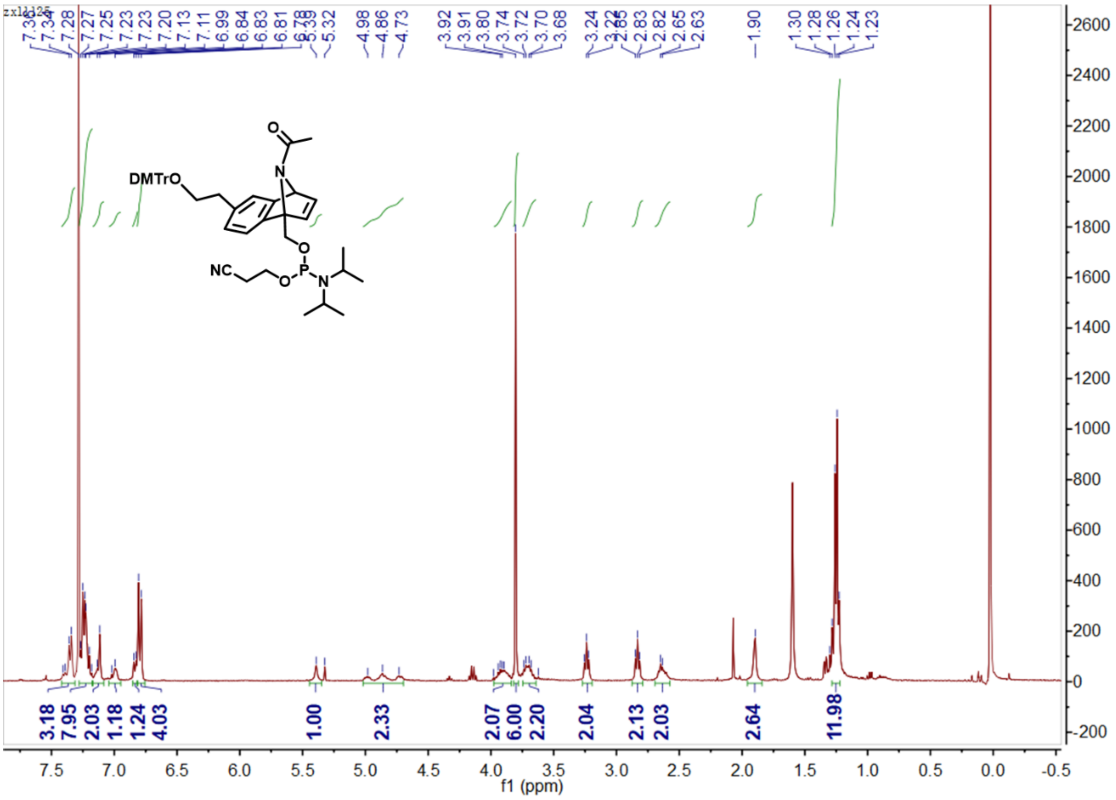

^{31}P NMR of **12**

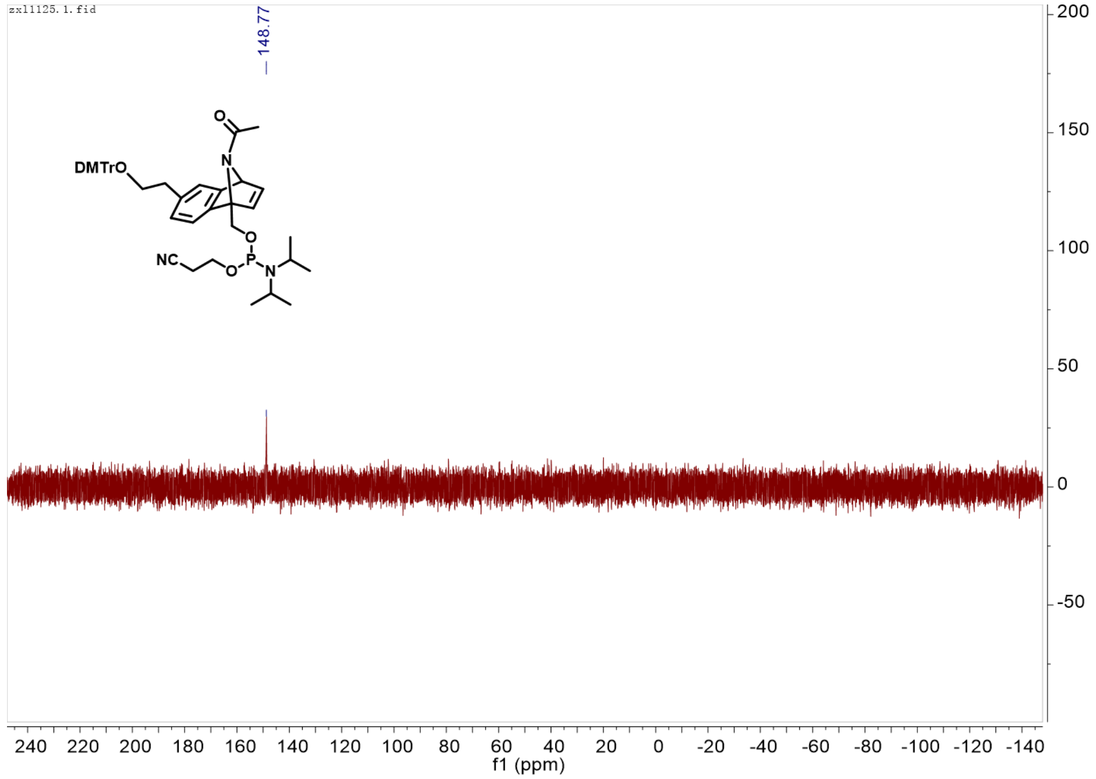

ESI-MS of compounds

ESI-MS of compound **6**

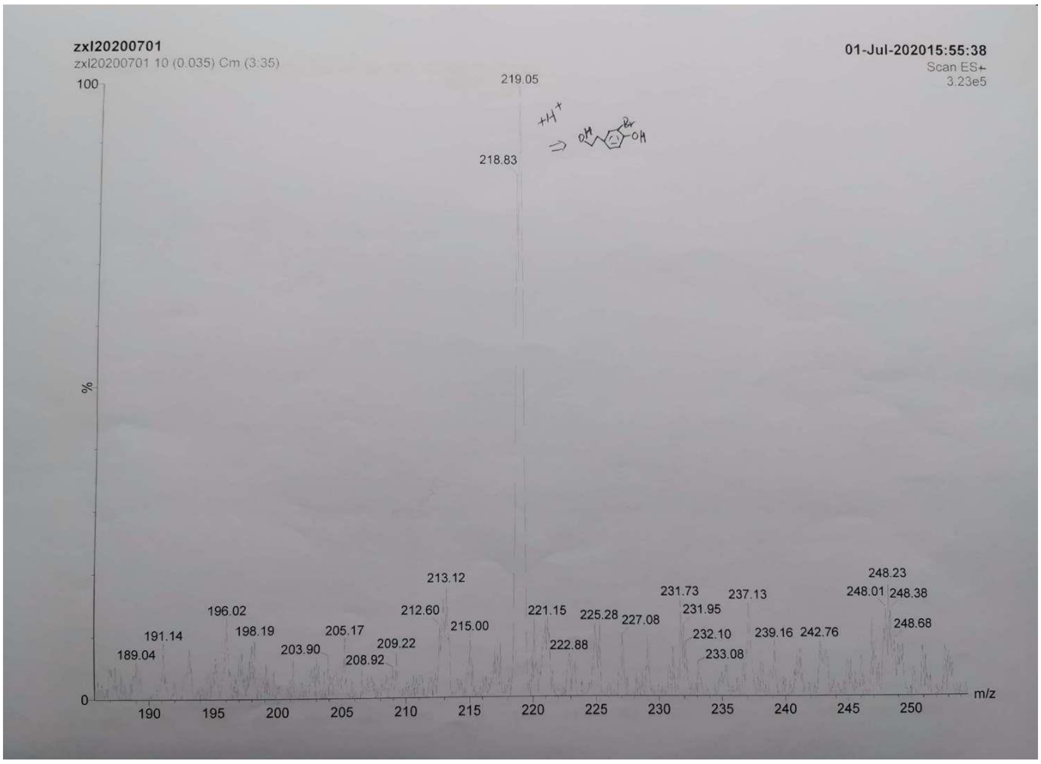

ESI-MS of compound **7**

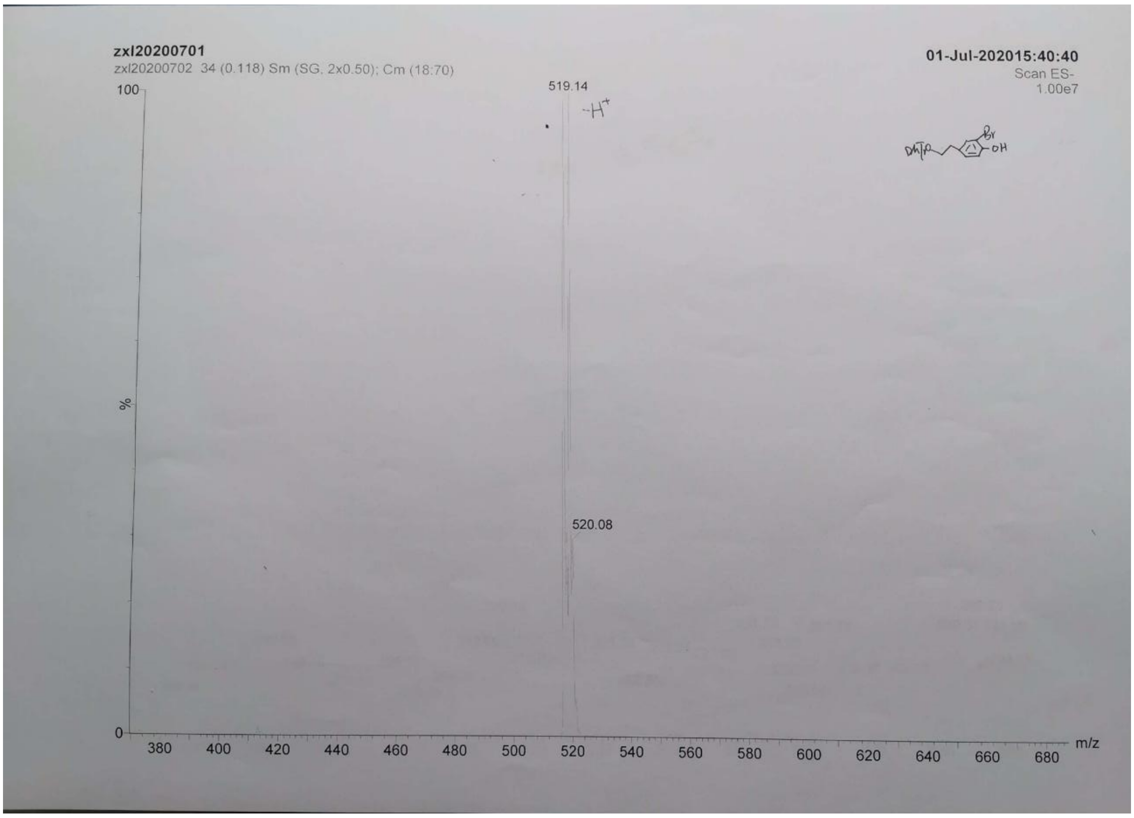

ESI-MS of compound **8**

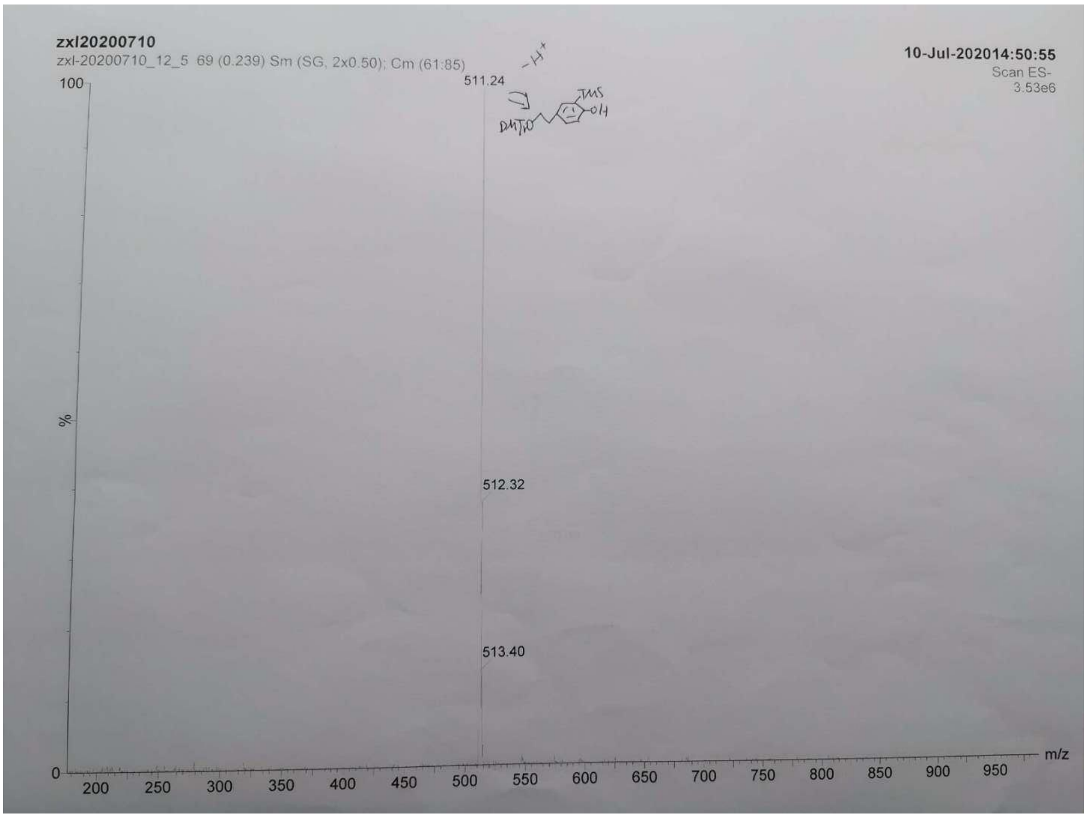

ESI-MS of compound **9**

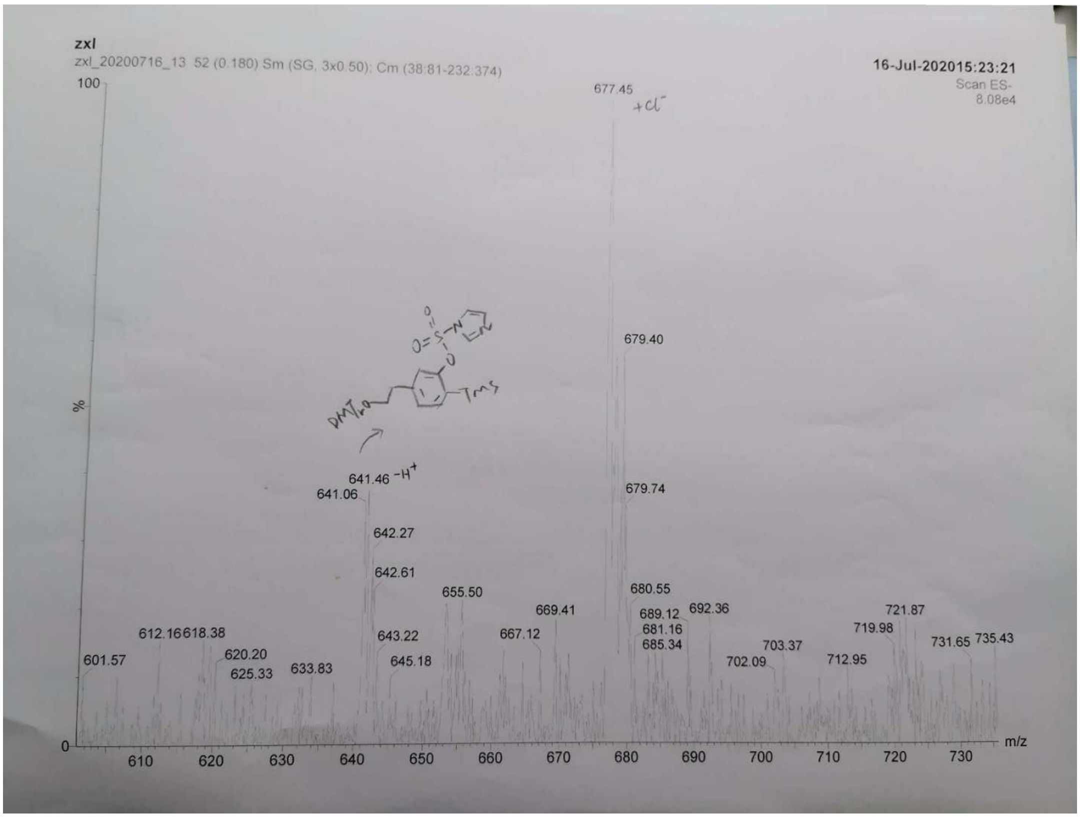

ESI-MS of compound **10**

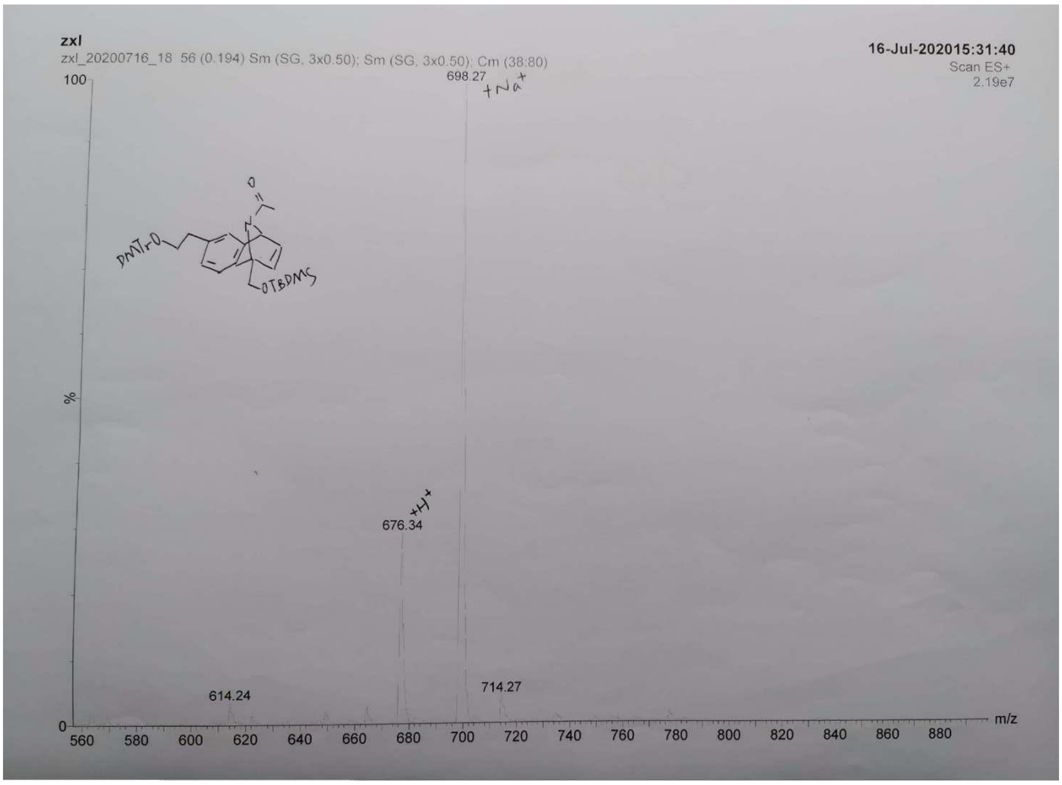

ESI-MS of compound **11**

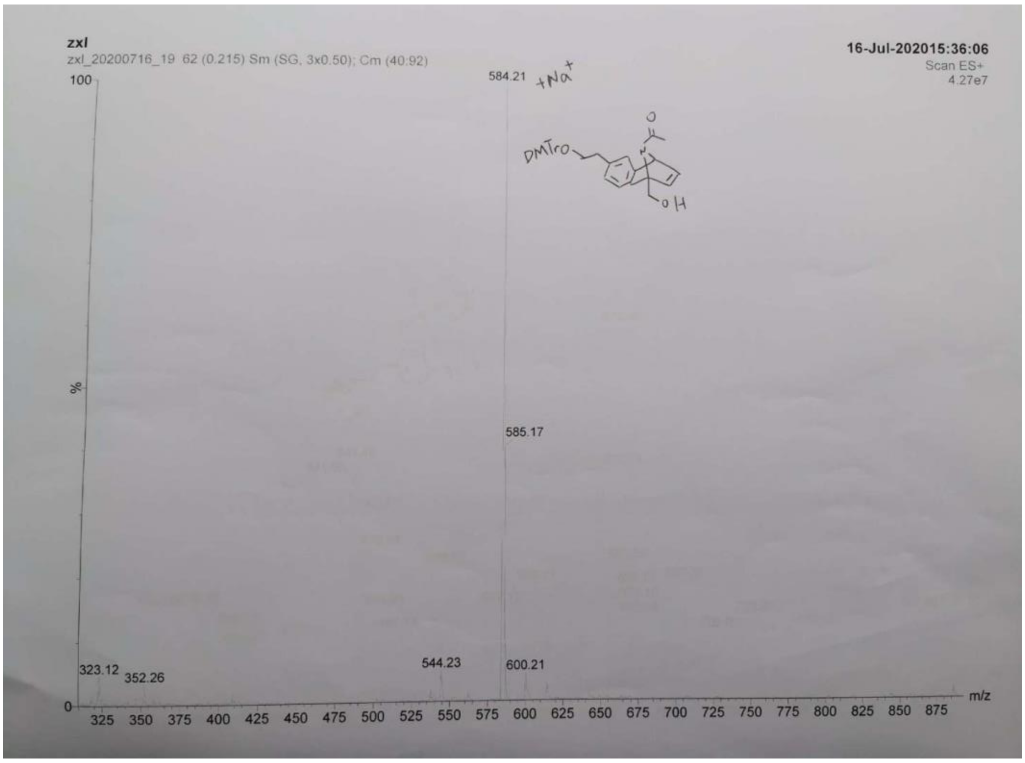

ESI-MS of modified oligonucleotides

VBAG

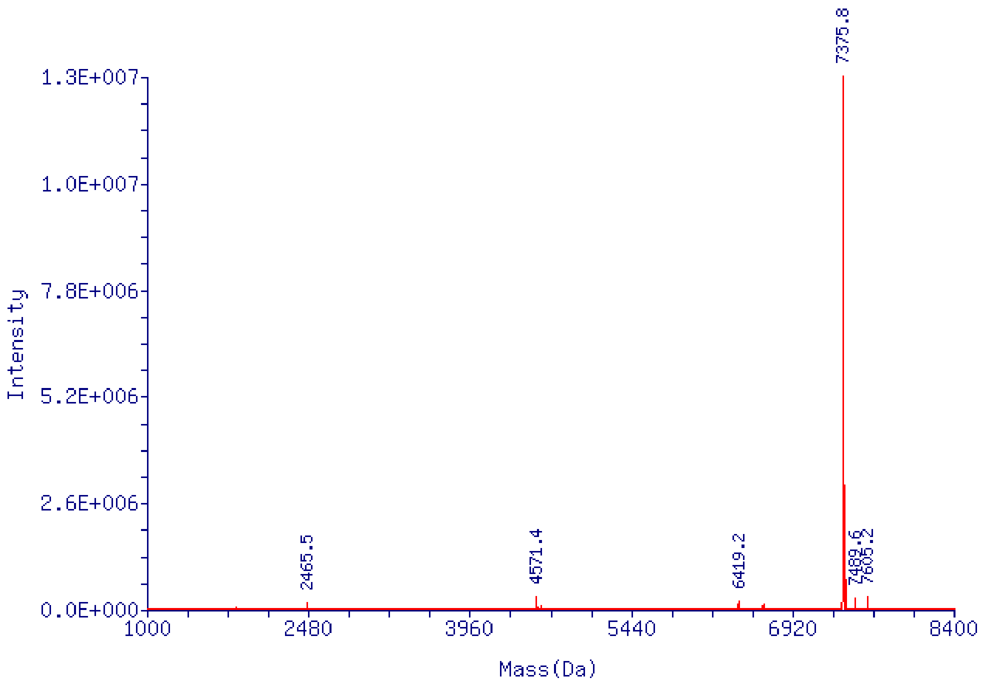

VBAG2

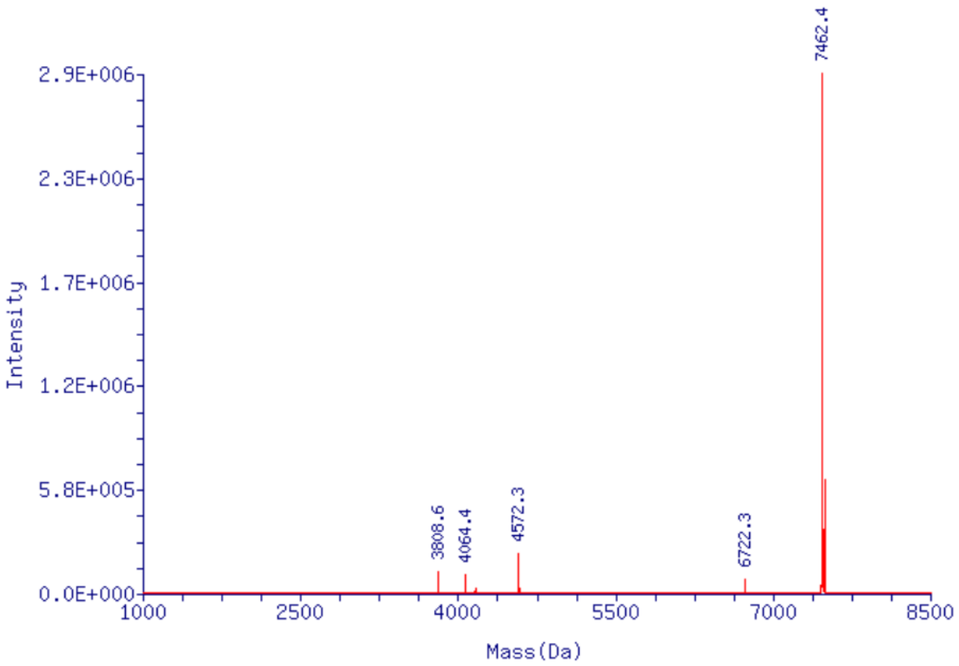
7462.4
2.9E+006
2.3E+006
1.7E+006
1.2E+006
5.8E+005
0.0E+000
Intensity
3808.6
4064.4
4572.3
6722.3
1000
2500
4000
5500
7000
8500
Mass(Da)

VAG2

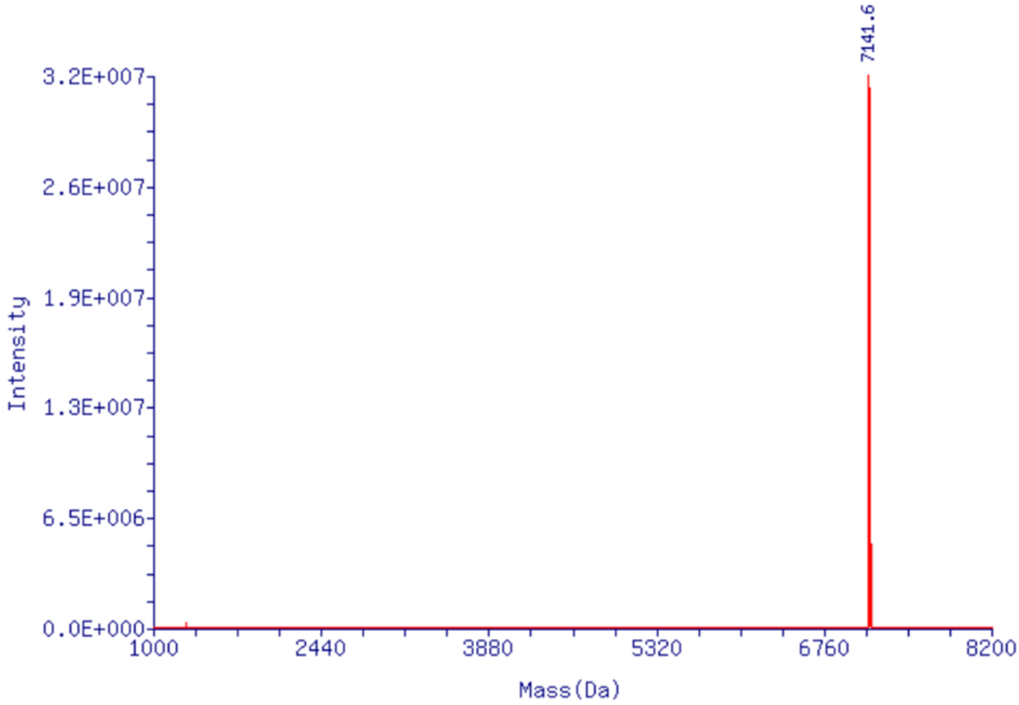
7141.6
3.2E+007
2.6E+007
1.9E+007
1.3E+007
6.5E+006
0.0E+000
Intensity
1000
2440
3880
5320
6760
8200
Mass(Da)

VBASE

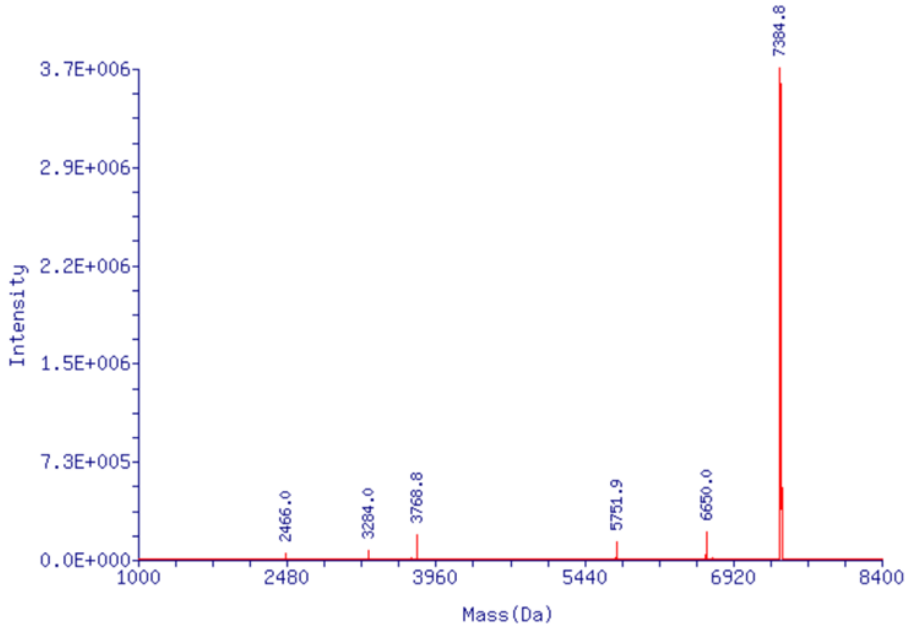
7384.8
3.7E+006
2.9E+006
2.2E+006
1.5E+006
7.3E+005
0.0E+000
Intensity
2466.0
3284.0
3768.8
5751.9
6650.0
1000
2480
3960
5440
6920
8400
Mass(Da)

VASE

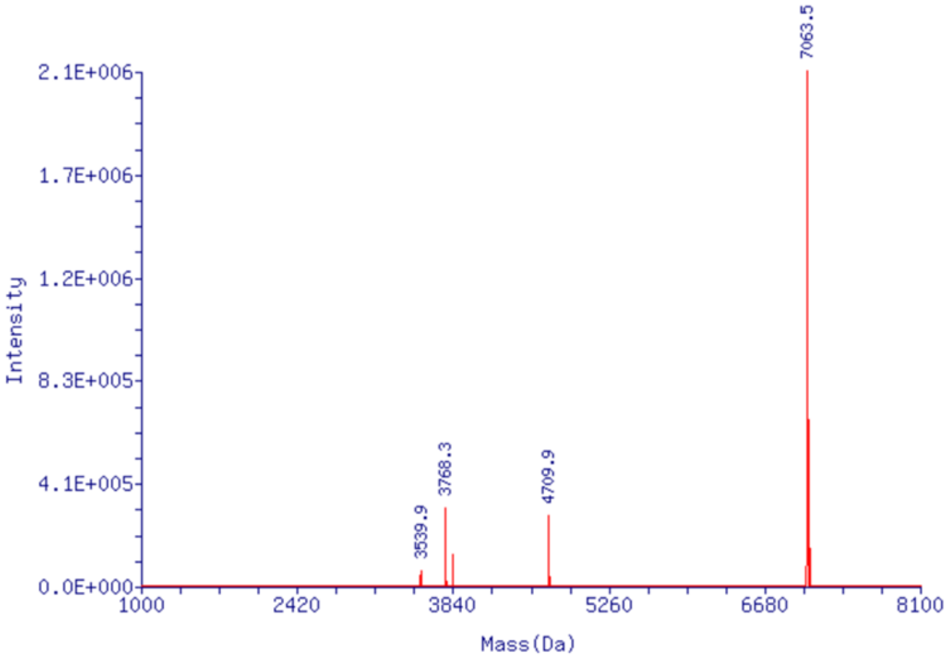
7063.5
2.1E+006
1.7E+006
1.2E+006
8.3E+005
4.1E+005
0.0E+000
Intensity
3539.9
3768.3
4709.9
1000
2420
3840
5260
6680
8100
Mass(Da)
